# Comparison of the Oral Microbiomes of Canines and Their Owners Using Next-Generation Sequencing

**DOI:** 10.1371/journal.pone.0131468

**Published:** 2015-07-02

**Authors:** Changin Oh, Kunkyu Lee, Yeotaek Cheong, Sang-Won Lee, Seung-Yong Park, Chang-Seon Song, In-Soo Choi, Joong-Bok Lee

**Affiliations:** Laboratory of Infectious Diseases, College of Veterinary Medicine and Veterinary Science Research Institute, Konkuk University, Seoul, 143-701, Republic of Korea; University of Illinois, UNITED STATES

## Abstract

The oral microbiome, which is closely associated with many diseases, and the resident pathogenic oral bacteria, which can be transferred by close physical contact, are important public health considerations. Although the dog is the most common companion animal, the composition of the canine oral microbiome, which may include human pathogenic bacteria, and its relationship with that of their owners are unclear. In this study, 16S rDNA pyrosequencing was used to compare the oral microbiomes of 10 dogs and their owners and to identify zoonotic pathogens. Pyrosequencing revealed 246 operational taxonomic units in the 10 samples, representing 57 genera from eight bacterial phyla. Firmicutes (57.6%), Proteobacteria (21.6%), Bacteroidetes (9.8%), Actinobacteria (7.1%), and Fusobacteria (3.9%) were the predominant phyla in the human oral samples, whereas Proteobacteria (25.7%), Actinobacteria (21%), Bacteroidetes (19.7%), Firmicutes (19.3%), and Fusobacteria (12.3%) were predominant in the canine oral samples. The predominant genera in the human samples were *Streptococcus* (43.9%), *Neisseria* (10.3%), *Haemophilus* (9.6%), *Prevotella* (8.4%), and *Veillonella* (8.1%), whereas the predominant genera in the canine samples were *Actinomyces* (17.2%), Unknown (16.8), *Porphyromonas* (14.8), *Fusobacterium* (11.8), and *Neisseria* (7.2%). The oral microbiomes of dogs and their owners were appreciably different, and similarity in the microbiomes of canines and their owners was not correlated with residing in the same household. Oral-to-oral transfer of *Neisseria shayeganii*, *Porphyromonas canigingivalis*, *Tannerella forsythia*, and *Streptococcus minor* from dogs to humans was suspected. The finding of potentially zoonotic and periodontopathic bacteria in the canine oral microbiome may be a public health concern.

## Introduction

The normal oral flora comprises numerous microorganisms. These microorganisms can either be protective and provide an essential barrier through interactions with the host immune system [1] or be pathogenic and cause diseases, such as dental caries, periodontitis, and systemic disease [2]. The composition of the oral microbiome can be altered by age, food consumption, environmental changes, and the health condition of the host [3]. When the host’s health deteriorates, some oral bacteria can spread to internal organs, resulting in an opportunistic infection [2]. However, the interactions of the various oral microbiome constituents with the host immune system and their potential pathogenicity are not clearly understood. To clarify these aspects, the Human Oral Microbiome project was undertaken, and the results are freely available in the Human Oral Microbiome Database (HOMD) [4].

Oral microbiome constituents, particularly bacteria that cause dental caries, can be transmitted from parents to their children through close physical contact [5]. Therefore, a guideline restricting direct oral contact between parents and their children has been introduced to reduce the prevalence of dental caries in children and adolescents [6]. Sometimes, the oral contact between dogs and their owners can be more extensive than that between parent and child. Dogs kiss and lick their owners to express amiable emotions. This raises the possibility of transferring bacteria from the oral microbiome of dogs to their human owners. In addition, periodontal diseases such as gingivitis and periodontitis are the most common diseases in dogs, with an estimated prevalence of 95–100% and 50–70%, respectively [7]. Several reports have described the companion animal-to-human passage of oral bacteria, including *Pasteurella multocida* and *Tannerella forsythia*, which are linked to local and systemic human infections [8][9]. A recent study on the distribution of oral periodontopathic bacterial species in dogs and their owners revealed that several periodontopathic species could be transmitted between humans and their companion dogs [10]. However, further studies using next-generation sequencing techniques, which can provide both broad and deep information about microbiomes, are needed to determine the relationship between the composition of the microbiomes of dogs and their owners.

In this study, we examined the oral microbiomes of dogs and their owners using next-generation sequencing technology. The similarity between the oral microbiomes of dogs and humans was assessed to evaluate the transmission of oral microorganisms between dogs and their owners. The taxa of the bacteria in the samples from the participants were determined to gain insight into the possibility of zoonotic pathogen transfer.

## Materials and Methods

### Ethics statement

The study was approved by the Konkuk University Institutional Animal Care and Use Committee (KU14017). The study was exempted from Institutional Review Board (P01-201401-BM-02-00) approval because the study design did not include any invasive procedures such as blood collection or the use of medication (S1 and S2 Files). Written informed consent was obtained from the human participants and dog owners to collect oral samples from them and their dogs, and the participants were informed that the samples would be anonymous. Therefore, we have no demographic or dental information for the human participants.

### Sample collection

Ten oral samples were collected from four pairs of dogs and owners as well as two humans from different households who had not kept a dog as a companion animal within the prior 10 years. The dogs had been spayed or neutered and were fed commercial diets free from raw or unpasteurized meat or milk products. The dogs were 3–5 years old (mean, 4 years) and were of small breeds: Dachshund (n = 1), Maltese (n = 1), Pomeranian (n = 1), and mixed breed (n = 1). The six human participants consisted of three males and three females aged 25–34 years (mean, 30.5 years). All dogs and humans were clinically healthy and had no history of surgery, anesthesia, or antimicrobial exposure within the preceding three months. The breed, age, household, and sex of each dog and the age, household, and sex of each human are shown in Table 1. Newly opened toothbrushes and plastic toothpicks were sterilized with ethylene oxide gas and ultraviolet light before sample collection. Oral samples were collected from three sites in the oral cavity, a buccal site, a palatal site, and the subgingival pouch. The buccal and palatal sites were brushed with a sterilized toothbrush for 30 s. The subgingival plaque was collected from the subgingival pouch of the left upper fourth premolar with a sterilized plastic toothpick. Immediately following collection, each toothbrush and toothpick was dipped into a 15-mL conical tube containing 2 mL of sterile TE buffer (10 mM Tris HCl, 1 mM EDTA; pH 8.0) and shaken vigorously to release the collected material. The sampled materials were transported at <4°C and processed within 24 h.

**Table 1:** Characteristics of the dogs and humans enrolled in this study.

Dogs & owners	Sample name	Household	Age	Sex	Breed	Tooth Brushing^b^	Closeness^c^
**Dog 1**	1_D	1	4	SF	Pom	3 times	4
**Human 1**	1_H	1	28	F			
**Dog 2**	2_D	2	5	SF	Dach	1 time	4
**Human 2**	2_H	2	25	F			
**Dog 3**	3_D	3	4	CM	Mal	1 time	3
**Human 3**	3_H	3	32	M			
**Dog 4**	4_D	4	3	SF	Mix	3 times	3
**Human 4**	4_H	4	32	M			
**Human 5**	C1^a^	Control_1	32	M			
**Human 6**	C2^a^	Control_2	34	F			

F: female, SF: spayed female, M: male, CM: castrated male, Pom: Pomeranian, Dach: Dachshund, Mal: Maltese, Mix: Mixed breed

^a^ Control human who had not raised a dog within the past ten years

^b^ Number of times per week teeth were brushed

^c^ Closeness score between dog and owner: 0, no contact, 1, nearly no contact with dog kept outdoors, 2, nearly no contact with dog kept indoors, 3, frequent contact without oral contact, 4, frequent contact with oral contact

### DNA extraction

Genomic DNA was extracted from each sample within 24 h of collection using the I-genomic DNA extraction mini kit (Intron, Seoul, Republic of Korea) according to the manufacturer’s bacterial DNA preparation protocol, with slight modifications. Briefly, place sampling TE buffer into a 2ml Eppendorf tube and mix vortexing vigorously. Then centrifuge at full speed for 5min at room temperature. Remove supernatant 150ul by pipetting and re-suspend pellet with remaining 50ul TE buffer. Add 200ul CG buffer, 20mg/ml of Proteinase K solution, 3ul RNase (10mg/ml) and mix vortexing. Then incubate the lysate at 65°C for 10~30min (Invert the lysate every 3 min). Add 250ul CB buffer and mix well by pipetting. Add 250ul of 80% ethanol into the lysate, and mix well by gently inverting 5–6 times or by pipetting. Do not vortex. Apply the mixture to spin column and centrifuge at 13,000rpm for 1min. Discard the filtrate and place the Spin Column in a new 2ml collection tube. Apply 700ul of CW buffer and centrifuge at 13,000rpm for 1 min, then again centrifuge for additionally 1 min to dry the membrane. Discard the flow-through and collection tube altogether. Place the spin column into a new 1.5ml tube, and 50ml of distilled water directly onto the membrane. Incubate for 2 min at room temperature and then centrifuge for 1 min at 13,000 rpm to elute.

### PCR amplification of bacterial 16S ribosomal RNA gene

Extracted DNA (20 ng) from each sample was PCR amplified according to the GS FLX library prep guide for library preparation. The 16S universal primers 27F (5′-GAGTTTGATCMTGGCTCAG-3′) and 518R (5′-WTTACCGCGGCTGCTGG-3ʹ) were used to amplify the 16S rRNA genes. The FastStart High Fidelity PCR System (Roche, Basel, Switzerland) was used for PCR under the following conditions: 94°C for 3 min followed by 35 cycles of 94°C for 15 s, 55°C for 45 s, and 72°C for 1 min, and a final elongation step at 72°C for 8 min.

### Library preparation

The PCR products were purified by using AMPure XP beads (Beckman Coulter, Palo Alto, CA). Libraries were quantified using the Picogreen assay (Victor 3). Emulsion-based PCR (emPCR) was carried out using the GS FLX emPCR Kit (454 Life Sciences, Branford, CT) for clonal amplification of the purified library. DNA capture beads were hybridized with single effective copies of template species from the DNA library to immobilize them for sequencing. The captured library was suspended in amplification solution and emulsified prior to PCR amplification. After amplification, the DNA-carrying beads were recovered from the emulsion and enriched. The second strands of the amplification products were melted away as part of the enrichment process, leaving the amplified single-stranded DNA library bound to the beads. The emulsion was dispensed into a 96-well plate, and the PCR amplification program was run according to the manufacturer’s recommendations.

### Next-generation sequencing using the Roche 454 GS FLX

Sequencing was performed by Macrogen (Seoul, Korea). Following PCR amplification, the emulsion was disrupted, and the beads carrying the amplified DNA library were recovered and washed by filtration. Positive beads were purified using the biotinylated primer A (complementary to adaptor A), which binds to streptavidin-coated magnetic beads. The DNA library beads were separated from the magnetic beads by melting the double-stranded amplification products, leaving a population of bead-bound single-stranded template DNA fragments. The sequencing primer was then annealed to the amplified single-stranded DNA. Finally, beads carrying amplified single-stranded DNA were counted with a Particle Counter (Beckman Coulter). Sequencing was performed with a Genome Sequencer FLX (454 Life Sciences), and each sample was loaded in 1/8 region of a 70 mm–75 mm Pico Titer plate (454 Life Sciences) fitted with an 8-lane gasket.

### Read trimming and taxonomic assignment

CD-HIT-OTU software was used for denoising and removing the homopolymer errors and chimeras. After filtering, CD-HIT-OTU generated operational taxonomic units (OTUs) by clustering sequenced data. Mothur software was used to analyze microbial communities [11]. OTUs were calculated to generate community diversity; Shannon diversity index [12] and Simpson index [13] were used to determine alpha diversity. Rarefaction analyses were conducted using the Mothur script “rarefaction.single”. Analysis of community similarity (beta diversity) was performed by calculating pairwise distances using the phylogenetic metric UniFrac [14]. All sequence reads were compared to the Silva rRNA database [15] using the Basic Local Alignment Search Tool (BLAST)N [16]. Similar sequence reads with E-values less than 0.01 were admitted as partial 16S rRNA sequences. Taxonomic assignment of the sequence reads was performed using the NCBI Taxonomy database. The taxonomy based on similarity was assigned according to these taxonomical guidelines: species, more than 97% similarity; genus, more than 94% similarity, family more than 90% similarity, order more than 85% similarity, class more than 80% similarity, and phylum more than 75% similarity [17]. The five most similar sequences for each read were determined by their bit scores and E-values using BLASTN. The Needleman-Wunsch global alignment algorithm was used to determine the optimum alignment of two sequences along their entire length. A pairwise global alignment was performed on selected candidate hits to find the best aligned hit [18]. The taxonomy of the sequence with the highest similarity was assigned to the sequence read.

## Results

### Analysis of pyrosequencing data

**Fig 1:**
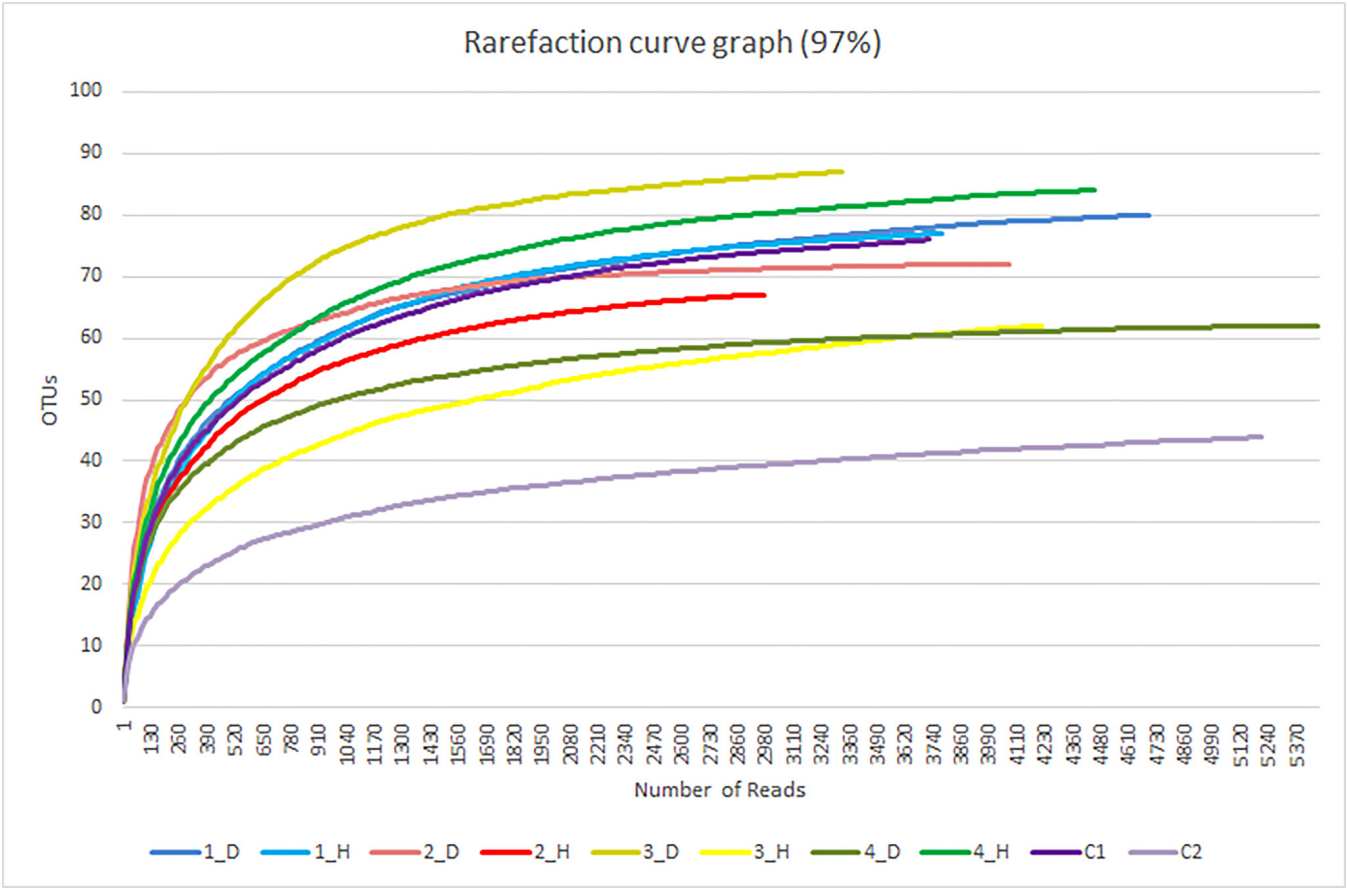
Rarefaction curve for 10 oral microbiome samples. Curves were plotted at a cutoff of 0.03 for each sample.

A total of 152,732 reads from the 10 samples were generated by high-throughput pyrosequencing. The mean number of sequences per sample was 15,273, and the average read length was 440.849. After removing sequencing errors and undesirable sequences using CD-HIT-OTU, a total of 41,821 reads remained, for an average of 4,182 reads per sample (Table 2). The rarefaction curve demonstrated good depth of coverage, with leveling of the curve by approximately 2,000 reads (Fig 1).

**Table 2:** The number of sequence reads and statistical analyses.

Sample	Raw data	Post-subsampling ^a^	OTUs	Richness ^b^	Shannon	Simpson
**1_D**	16289	4688	80	81.8	3.14	0.07
**1_H**	13434	3767	77	79.3	2.52	0.2
**2_D**	16802	4068	72	72	3.58	0.04
**2_H**	12668	2963	67	70	3.09	0.08
**3_D**	13437	3313	87	90	3.37	0.06
**3_H**	14204	4204	62	69.3	2.53	0.12
**4_D**	16958	5452	62	62.2	2.95	0.09
**4_H**	18920	4445	84	88.7	3.08	0.09
**C1**	15559	3714	76	80.5	3.06	0.08
**C2**	14461	5207	44	51	1.98	0.23
**Total**	152732	41821	246^c^	-	-	-
**Mean**	15273.2±1869	4182.1±751	71.1±12.1	74.5±11.4	2.93±0.44	0.1±0.06
**Mean (Human)**	14874.3±2016	4050±694	68.3±12.9	73.1±11.9	2.71±0.41 ^d^	0.14±0.06 ^e^
**Mean (Dog)**	15871.5±1427	4380.3±787	75.25±9.3	76.5±10.4	3.26±0.24 ^d^	0.06±0.02 ^e^

Total number of sequences, indices for the number of observed operational taxonomic units (OTUs) per sample, diversity, and richness in 10 oral samples

^a^ Trimming and chimera removal

^b^ As calculated with Chao 1

^c^ Total OTUs in all 10 samples

^d & e^ They were significant differences (*p*< 0.05).

### OTU based analysis

**Fig 2:**
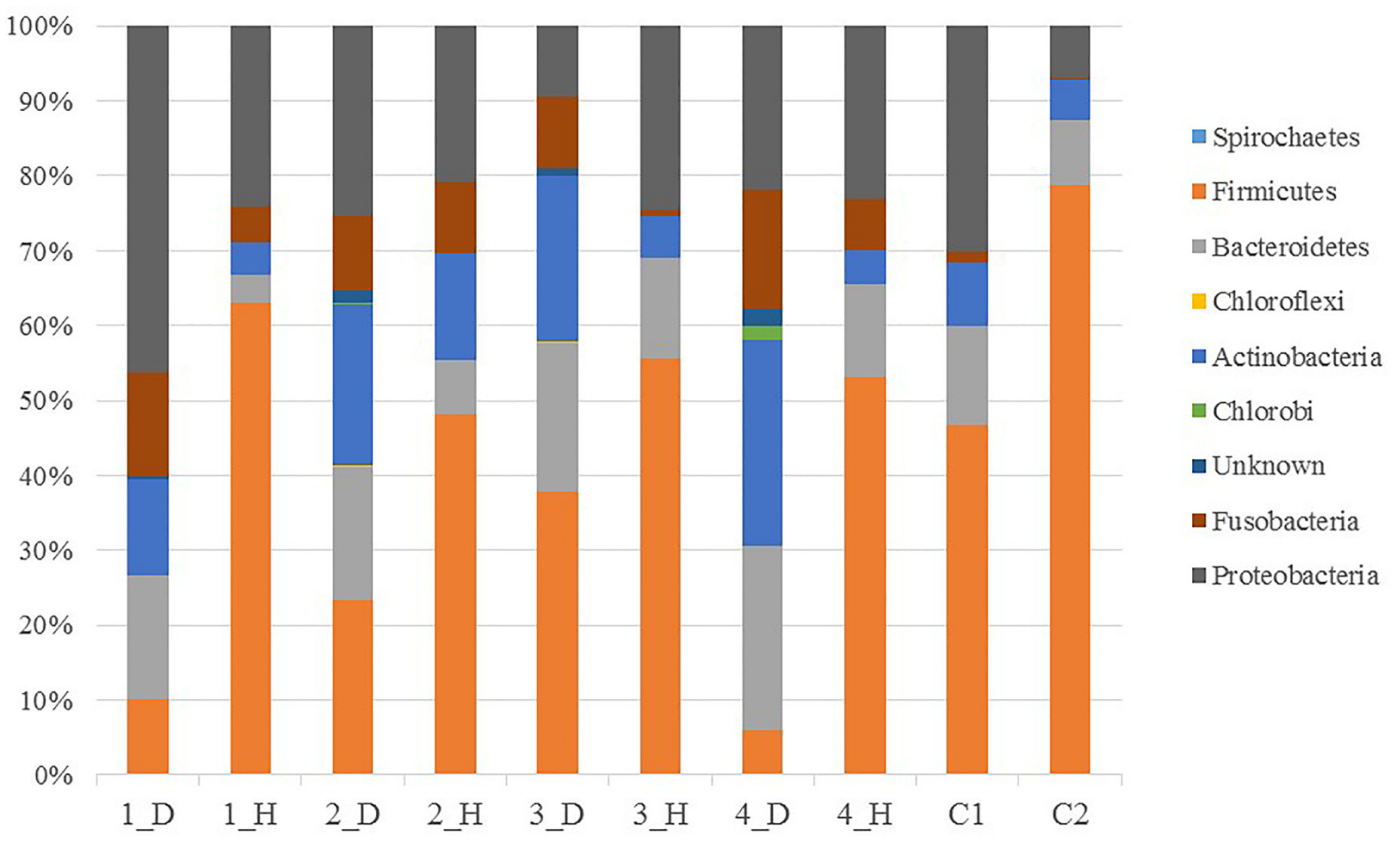
Relative distribution of sequences in the OTUs of the 10 oral samples. Phyla corresponding to more than 0.5% of the sequences determined from the mean distribution of phyla.

**Fig 3:**
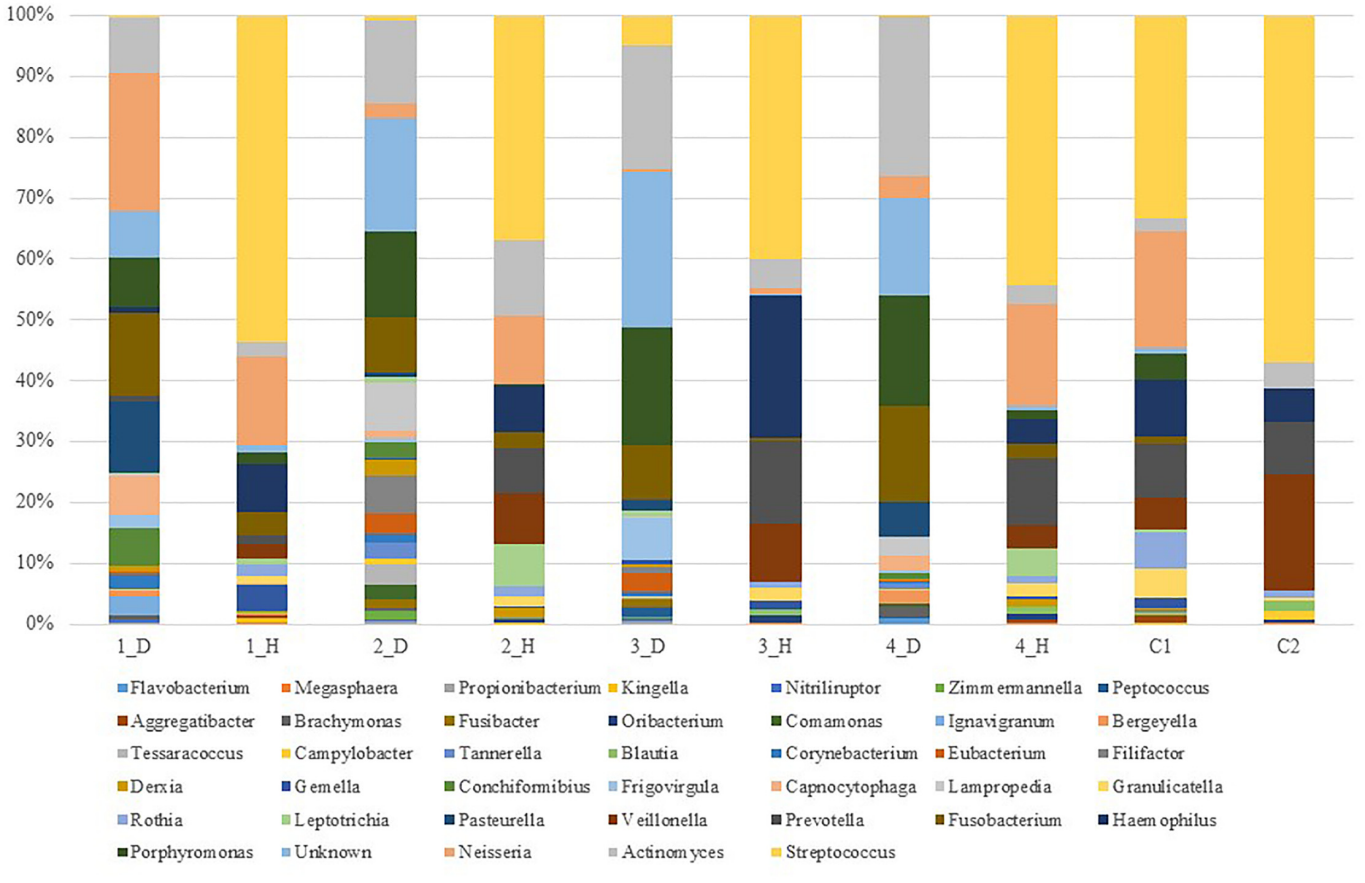
Relative distribution of sequences in the OTUs in 10 oral samples. Distribution at the genus level.

Sequences were clustered into 246 OTUs, with an average of 71.1±12.1 OTUsper sample (Table 2). Eight known phyla were represented among the 246 OTUs. The consensus taxonomy, which was assigned using the Silva database, indicated that each OTU belonged to the domain Bacteria and to one of eight phyla. In the human oral samples, five phyla had relative sequence abundances greater than 1%: Firmicutes (57.6%), Proteobacteria (21.6%), Bacteroidetes (9.8%), Actinobacteria (7.1%), and Fusobacteria (3.9%). In the canine oral samples, six phyla had relative abundances greater than 1%: Proteobacteria (25.7%), Actinobacteria (21%), Bacteroidetes (19.7%), Firmicutes (19.3%), Fusobacteria (12.3%), and Unknown (1.3%) (Fig 2). A total of 57 genera were represented by the OTUs. In the human oral samples, the bacterial taxa with >1% abundance were *Streptococcus* (43.9%), *Neisseria* (10.3%), *Haemophilus* (9.6%), *Prevotella* (8.4%), *Veillonella* (8.1%), *Actinomyces* (4.8%), *Rothia* (2.1%), *Leptotrichia* (2.1%), *Granulicatella* (2.1%), *Fusobacterium* (1.7%), *Gemella* (1.3%), and *Porphyromonas* (1.3%). In the canine oral samples, the bacterial taxa with >1% abundance were *Actinomyces* (17.2%), Unknown (16.8), *Porphyromonas* (14.8), *Fusobacterium* (11.8), *Neisseria* (7.2%), *Pasteurella* (4.9%), *Lampropedia* (2.8%), *Capnocytophaga* (2.5%), *Frigovirgula* (2.5%), *Conchiformibius* (2.4%), *Filifactor* (1.8%), *Eubacterium* (1.7%), *Streptococcus* (1.5%), *Corynebacterium* (1.2%), and *Derxia* (1.1%) (Fig 3).

### Statistical analyses

**Fig 4:**
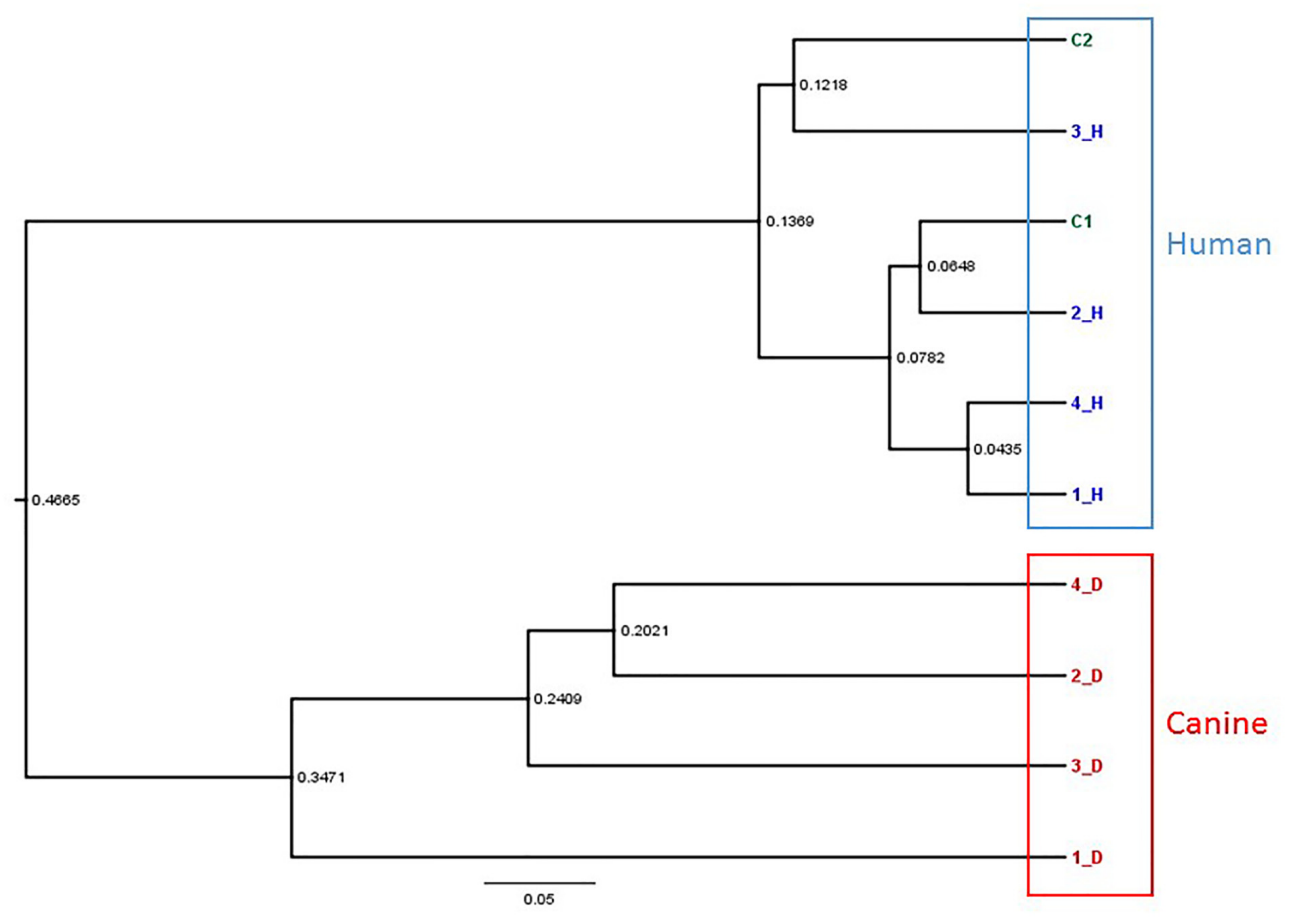
Phylogenetic tree showing the similarity in the oral microbiota of canines and their owners based on the neighbor-joining methods.

**Fig 5:**
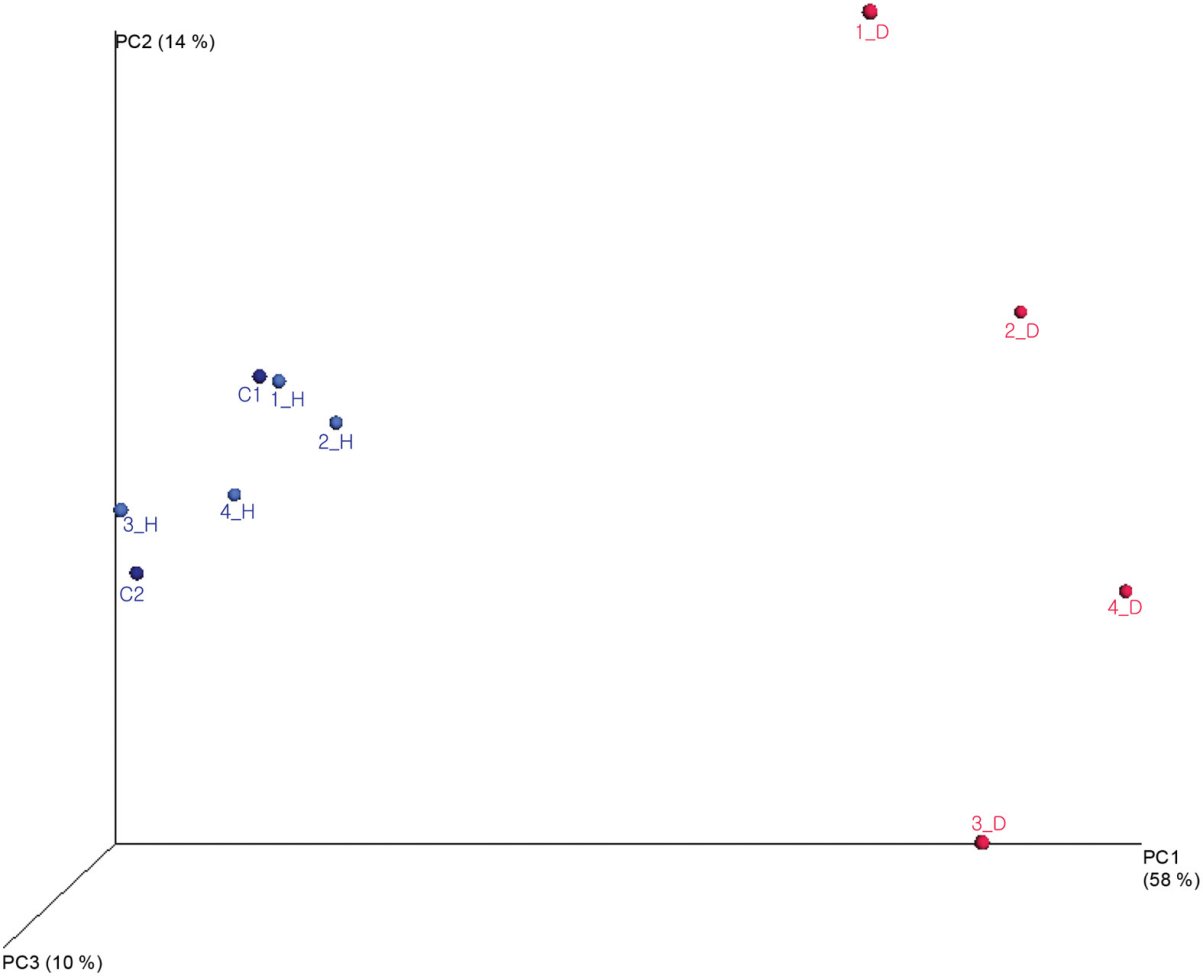
Three-dimensional principal coordinate analysis (PCoA) plot of samples using the weighted UniFrac distance metric. Percentage of the diversity distribution explained by each axis is indicated on the fig.

The diversity, richness, evenness, and means of each value in dogs and humans and the number of OTUs are presented in Table 2. The average richness of the human oral samples (73.1±11.9) was lower than that of the canine samples (76.5±10.4). This result indicated that the canine oral microbiome was richer than the human oral microbiome but it was not statistically significant (p-value = 0.6469). Comparison of the average alpha diversity indices (Shannon, Simpson) for human and canine samples revealed that the canine oral microbiome was more diverse than the human oral microbiome. The similarity of the human and canine oral microbiomes in the 10 oral samples was demonstrated using a phylogenetic tree generated by the neighbor-joining method (Fig 4). In the phylogenetic tree, the bacterial species in the canine and human oral microbiomes clustered together, this clustering was independent of whether the dog and human were from the same household. The intraspecies similarity of the oral microbiomes was higher than the intra-household similarity of the oral microbiomes. The similarity in the microbial communities of the canine samples was compared to that of the owners’ samples by the weighted UniFrac distance metric (Fig 5). The weighted UniFrac distance pairs in house 1 and 2, were lowest among the owners. However, the weighted UniFrac distance pairs in house 3 and 4 were not the lowest pairs. This result indicated that the oral microbiomes of dog 1 and dog 2 were the most similar to the oral microbiomes of their owners, but the oral microbiomes of dog 3 and dog 4 were most similar to that of the other owners.

### Bacterial taxa found in both the human and canine oral microbiomes

Among the 246 OTUs from the 10 oral samples, 12 OTUs (12/246, 4.8%) were found in both human and canine oral samples (Table 3). All sequences were assigned to three different databases: SILVA, HOMD, and NCBI. The sequences shared 99–100% similarity with the reference sequences in the NCBI BLAST database [16]; however, the similarity scores were lower for the other database searches. Four OTUs seem to be related with spread of oral bacteria between dogs and humans. *Neisseria shayeganii* was found in the samples of all four dog owners and dogs, but not in the control individuals (C1, C2). *Porphyromonas canigingivalis*, *Tannerella forsythia*, and *Streptococcus minor* were found in all canine samples. *Porphyromonas canigingivalis* was only found in the sample from the human in household 4 (4_H), and *Tannerella forsythia* and *Streptococcus minor* were only found in the sample from the human in household 3(3_H) (Table 1).

**Table 3:** Bacteria found in the oral samples from both dogs and owners.

SILVA	Identity	HOMD	Identity	NCBI	Identity	1_D	1_H	2_D	2_H	3_D	3_H	4_D	4_H	C1	C2
Fusobacterium sp.	98%	Fusobacterium nucleatum	97.6%	Fusobacterium sp.	99%	6.7	0	51	0	30	0.9	9.9	0	1.1	0.2
Fusobacterium sp.	99%	Fusobacterium periodonticum	99.8%	Fusobacterium sp.	99%	0.4	31.3	1.1	18.4	1.6	4.2	12.9	24.2	5.3	0.4
Porphyromonas cangingivalis	99%	Porphyromonas endodontalis	86%	P. cangingivalis	99%	17.5	0	19.5	0	24.8	0	38.1	0.05	0	0
Filifactor alocis	100%	Filifactor alocis	100%	Filifactor alocis	100%	0	0	0	0	16.7	0	0	0	83.3	0
Uncultured bacterium	99%	Neisseria weaveri	94.3%	Neisseria shayeganii	99%	81.8	1.9	13.1	0.7	0.1	0.1	1.9	0.4	0	0
Uncultured bacterium	99%	Neisseria flavescens	98.9%	Uncultured Neisseria sp.	99%	0	4.8	0	52.1	0	0	0.3	32.1	10.6	0
Tannerella forsythensis	100%	Tannerella forsythia	97.2%	Tannerella forsythia	100%	1.2	0	64.8	0	3.7	0.6	29.6	0	0	0
Streptococcus minor strain	100%	Streptococcus sinensis	91.4%	Streptococcus minor	100%	5.6	0	0	0	92.5	1.3	0.6	0	0	0
Uncultured bacterium	99%	Lautropia mirabilis	98.5%	Lautropia sp.	100%	15.6	3.5	35.9	16.8	4.1	0.3	3.5	17.5	2.9	0
Actinomyces sp.	100%	Actinomyces cardiffensis	99.5%	Actinomyces cardiffensis	100%	0	0	0	0	66.7	0	0	0	33.3	0
Sequence 6 from PatentWO2008137541	100%	Peptostreptococcus anaerobius	85.4%	Frigovirgula sp.	100%	31	0	0	0	60.8	0	7.9	0.3	0	0
Uncultured Lachnospiraceae bacterium	96%	Lachnospiraceae [G-8] sp.	95.5%	Lachnospiraceae bacterium	99%	5.28	0	0.8	0	82.3	0	10.2	0	1.5	0

SILVA: Silva ribosomal RNA database, HOMD: Human oral microbiome database, NCBI: NCBI basic local alignment search tool database, Identity: Identity is the percent similarity between the query and subject sequences over the length of the coverage area. The numbers below the sample names were correspond to the relative abundance (sample reads/total reads×100) of the read number of the samples in each OTU.

### Opportunistic pathogens and periodontopathic bacteria in the canine oral microbiome

The OTUs exclusively in the canine oral samples are listed in Table 4. Among the OTUs, those known or suspected to be zoonotic [19] are described below. *Pasteurella dagmatis* canine oral taxon 092 clone OE001 (602 reads) and *Pasteurellaceae bacterium* canine oral taxon 080 clone OC053 (337 reads) were identified in the canine oral samples. Human pasteurellosis is often caused by dog or cat bites, resulting in cellulitis and subcutaneous abscesses. *Pasteurella* spp. infrequently cause systemic infectious diseases and mostly infect patients with underlying diseases [20]. *Capnocytophaga cynodegmi* canine oral taxon 254 clone ZX121, the causative agent of bite wound infections [21], was also identified (485 reads).

**Table 4:** Bacteria found only in canine oral samples.

NCBI blast result	Identity^a^	1_D	2_D	3_D	4_D	Total Reads^b^	%^c^
*Actinomyces* sp. COT 083 clone OC035	99%	8.7	21.5	10.9	58.8	1685	13.1
*Fusobacterium* sp. COT 189 clone QD044	97%	38	8	7.7	46.2	1599	12.2
*Actinomyces canis* strain CCUG 41706	99%	7.9	15.7	39.5	36.9	998	7.6
*Pasteurella dagmatis* COT 092 clone OE001	99%	85.9	0	1.8	12.3	602	4.6
*Moraxella* sp. canine oral taxon 017 clone OH079	99%	2	7.9	36.5	53.7	598	4.6
*Erysipelotrichaceae bacterium* COT 311 clone ZY009	99%	25	5.3	32.2	37.4	583	4.5
*Xenophilus* sp. COT	98%	0.2	64.8	1.4	33.5	489	3.7
*Capnocytophaga cynodegmi* COT 254 clone ZX121	99%	63.3	9.3	1.2	26.2	485	3.7
*Neisseria weaveri* COT 269 clone ZL078	100%	62.1	0	0	37.9	480	3.7
*Pasteurellaceae bacterium* COT 080 clone OC053	99%	11.3	8	11.6	69.1	337	2.6
*Filifactor villosus* COT 031 clone OD049	99%	0	87.7	11.2	1.1	285	2.2
*Conchiformibius steedae* COT 280 clone ZP010	97%	89.5	0	0	14.4	267	2.
*Peptostreptococcaceae bacterium* COT 047 clone OD006	100%	7.4	52.3	37.2	3.1	258	2
*Porphyromonas* sp. COT	99%	2.6	5.7	41.9	49.8	227	1.7
*Lachnospiraceae bacterium* COT 073 clone Zi333	100%	2.8	71.6	20.9	4.7	211	1.6
*Actinomyces* sp. COT 252 clone ZI340	99%	14.6	18.5	29.8	37.1	178	1.4
SR1 bacterium COT 369 clone 2B042	100%	6	9	19.9	68.7	166	1.3
*Conchiformibius* sp. COT 286 clone ZQ020	99%	33.9	63.7	0	0	164	1.3
*Actinomyces* sp. COT 374 clone 2B067	96%	97.4	0	0	2.6	156	1.2
*Porphyromonas gulae* COT 052 clone QC036	100%	0	89.7	10.3	0	155	1.2
*Globicatella* sp. COT 107 clone OH001	100%	92.7	0	3.3	4	151	1.2
*Neisseria zoodegmatis* COT 349 clone 1S040	98%	100	0	0	0	149	1.1
*Porphyromonas gingivicanis* COT 022	100%	6.6	26.3	8.0	59.1	137	1
*Brachymonas* sp. COT 015 clone OB002	100%	25.7	11	0.7	62.5	136	1

The numbers below the sample names were correspond to the relative abundance (sample reads/total reads×100) of the read number of the samples in each OTU. COT is abbreviation of Canine oral taxon.

^a^ Identity, which is the percent similarity between the query and subject sequences over the length of the coverage area.

^b^ Total reads refer the sum of read in each OTU.

^c^ % is the relative abundance of the total reads in bacteria found only in canine oral sample.

Periodontopathic bacteria were detected in all sampled dogs. *Actinomyces* sp. canine oral taxon 083 clone OC035 (1,685 reads), *Actinomyces canis* strain CCUG 41706 (998 reads), *Actinomyces* sp. canine oral taxon 252 clone ZI340 (178 reads), and *Actinomyces* sp. canine oral taxon 374 clone 2B067 (156 reads), the causative agents of actinomycosis, periodontal disease, and endocarditis, respectively [22],[23] were the most frequently identified taxa in the canine oral samples. *Fusobacterium* sp. canine oral taxon 189 clone QD044 (1,599 reads), which causes periodontitis and infects dog and cat bite wounds [24], was the second most abundant species in the canine oral microbiome samples. Two Neisseria species were identified, *Neisseria weaveri* canine oral taxon 269 clone ZL078 (480 reads) and *Neisseria zoodegmatis* canine oral taxon 349 clone 1S040 (164 reads); both are opportunistic pathogens in human peritonitis, lower respiratory tract infections, wound infections, septicemia, and periodontitis [25]. *Peptostreptococcaceae bacterium* canine oral taxon 047 clone OD006 (258 reads), the causative agent of periodontitis, is a member of the orange complex [26]. *Porphyromonas* sp. canine oral taxon (227 reads), *Porphyromonas gulae* canine oral taxon 052 clone QC036 (155 reads), and *Porphyromonas gingivicanis* canine oral taxon 022 (137 reads) are associated with periodontal disease. *Porphyromonas gulae*, a major cause of periodontitis in dogs [27], closely resembles *Porphyromonas gingivalis*, the primary periodontal pathogen in humans [28][29].

## Discussion

High-throughput pyrosequencing was used to determine the diverse taxa present in 10 oral samples from dogs and humans. The results differed somewhat from those in a previous study of the canine oral microbiome [30]. The number of identified OTUs in this study (246) was lower than that of the previous study. In addition, there were differences in the genus-level distributions. In the former study, *Porphyromonas* (39.23%), *Fusobacterium* (4.53%), *Capnocytophaga* (3.78%), and *Derxia* (3.70%) were the four most abundant genera. However, in this study, *Porphyromonas* (14.8%), *Fusobacterium* (11.8%), *Actinomyces* (17.1%), *Neisseria* (7.2%), and *Pasteurella* (4.9%) were the most abundant genera. The reasons for the differences include variations in the oral microbiome composition of the enrolled dogs, differences in the sample collection method, and differences in the trimming and denoising processes.

The results of the present study suggest that bacterial dissemination between dogs and human is uncommon. This result contradicts previous findings [10]. The contrasting results of the two studies can be attributed to the difference in the detection methods. In the previous study, pathogens were detected by PCR and gel electrophoresis; however, in this study, we used next-generation sequencing, which is more accurate. It is possible that the bacteria found in both human and canine samples in the previous study were actually different bacterial species and false positives.

Few bacteria from the canine oral microbiome were found in the owners’ oral samples. The number of OTUs identified in both dogs and humans (4.9%, 12/246), was much lower than that in the previous study [31]. Furthermore, among these shared OTUs, only four seemed to have disseminated from dogs to humans. In addition, these shared OTUs were of relatively lower prevalence. For example, *Porphyromonas canigingivalis*, which was found in both canines and owners, accounted for 17.5%, 19.5%, 24.8%, and 38.1% relative abundances in the four canine oral samples, but was detected as only one read in a single human sample (4_H). The low number of reads for the shared OTUs indicates that these bacteria are either few in number or were transmitted but failed to colonize the human oral cavity.

To evaluate the influence of close contact in the same household, a phylogenetic tree and UniFrac distance were used. In the phylogenetic tree, the 10 oral microbiome samples clustered by species rather than by household. This result indicated that species differences were more influential than close contact in the same household. In the UniFrac distance analysis, the oral microbiome compositions of two dogs (1_D, 2_D) were most similar to those of their owners’; however, the oral microbiomes of two other dogs (3_D, 4_D) were highly similar to those of humans who were not their owners. The similarities between the microbiomes of dogs and humans were not correlated with household. This result can be explained by the closeness score. Since households 1 and 2 had closeness scores of 4, which indicated frequent oral contact, the oral microbiomes of the dogs were the closest to those of the human within their household. Although the owners in households 3 and 4 were in frequent contact with their dogs, their oral microbiomes were not highly similar to those of their dogs because they did not have oral contact. This result implies that oral microbiomes are more influenced by oral-to-oral contact than by oral-to-skin and skin-to-skin contact.

It is difficult for canine oral bacteria to survive and colonize the human oral environment because of the physiological differences between the two environments. First, dog saliva is less acidic (pH 8.5–8.65) than that of human saliva (pH 6.5–7.5) [32]. Because the oral microbiome is altered by pH [33], most bacteria in the dog oral cavity may not survive well in the human oral environment. Second, most humans brush their teeth every day. During tooth brushing, most of the supra-gingival bacteria are removed and are then regenerated. Since dogs teeth are typically brushed less frequently than those of humans, they usually have a mature biofilm that is substantially different from the immature human oral microbiome. When canine oral bacteria are transmitted to the human oral cavity, they have to compete with human oral microbiome constituents and are removed by frequent tooth brushing. The present study corroborated these differences by comparing the microbial composition at the genus level. In the human oral samples, the most abundant genus was *Streptococcus*, which comprises organisms that are early colonizers in oral biofilm formation. Conversely, in the oral samples from dogs, anaerobic late colonizers, including *Actinomyces*, *Porphyromonas*, and *Fusobacterium*, were the most abundant genera, which are periodontopathic bacteria characterized by their proteolytic activity [1][34].

This study has several limitations. First, the affinity of the primer set was different for each bacterium. This primer bias is considered an inevitable problem when using 16S rRNA. Several universal primer sets were used to overcome this primer bias. Second, the results could vary depending on the analysis tools used. Every analysis tool has its advantages and drawbacks. Therefore, a gold standard analysis method needs to be established for metagenomics. Third, the taxonomic assignments varied according to the database used. When using the Silva database and the human oral microbiome database, some sequences in the OTUs were not assigned to any specific genus; however, when these were compared to sequences in the NCBI database using BLAST, matches with greater than 97% identity were found. Although, Silva and HOMD are specific to 16S rRNA, they require constant updating for more accurate assignments. Finally, the number of participants was too low to derive statistical significance. Therefore, more in-depth studies including large numbers of participants are required to reveal the causal relationship of periodontopathic bacteria and their transmission between dogs and their owners.

In this study, bacterial transmission from dogs to humans was found to be limited. Although oral bacteria are not contagious, the canine oral cavity might harbor potential zoonotic pathogens. The dog owners and people who are involved in the pet industry should be aware of potential hazard of dog oral bacteria. To reduce the numbers of potential pathogens and possibility of accidental infection, regular tooth brushing and dental scaling are recommended.

## Supporting Information

S1 File Certification of exemption from IRB. (PDF)

S2 File Exemption from IRB self check list. (PDF)
