# Supplementary material for: Comparison of the Oral Microbiomes of Canines and Their Owners Using Next-Generation Sequencing
Source: PLoS One. 2015 Jul 2;10(7):e0131468. doi: 10.1371/journal.pone.0131468 (PMC4489859; doi:10.1371/journal.pone.0131468)
Supplement: S1 File — (PDF) [file pone.0131468.s001.pdf]

심의면제확인서

이 중 복　귀하

**과제관리번호**	P01-201401-BM-02-00					
**연구과제명**	반려견주와 반려견간 구강내 세균총의 비교					
**연구책임자**	성명	이중복	소속	건국대학교	직위	교수

상기 연구과제에 대하여 본 위원회에서는 심의면제대상임을 확인합니다.

※ 모든 연구자들은 아래의 사항을 준수하여야 합니다.

1) 계획서에 따라 연구를 수행하여야 합니다.
2) 위원회의 요구가 있을 때에는 연구의 진행과 관련된 보고를 위원회에 제출하여야 합니다.
3) 연구윤리를 위하여 관련부처가 필요시 조사 및 감독 차원에서 현장점검을 실시할 수 있습니다.
4) 연구와 관련된 기록은 연구가 종료된 시점을 기준으로 최소 3년간 보관하여야 합니다.

2014년 1월 15일

보 건 복 지 부 지 정

공 용 기 관 생 명 윤 리 위 원 장 (인)

본 확인서에 기재된 사항은 보건복지부 지정 공용기관생명윤리위원회에 기록된 내용과 일치함을 증명합니다.
본 공용기관생명윤리위원회는 생명윤리 및 안전에 관한 법률과 관련 법규를 준수합니다.
본 연구와 이해상충(Conflict of Interest)이 있는 위원이 있을 경우 연구의 심의에서 배제합니다.
본 확인서의 사본은 공용기관생명윤리위원회에서 보관합니다.
