# Supplementary material for: Comparison of the Oral Microbiomes of Canines and Their Owners Using Next-Generation Sequencing
Source: PLoS One. 2015 Jul 2;10(7):e0131468. doi: 10.1371/journal.pone.0131468 (PMC4489859; doi:10.1371/journal.pone.0131468)
Supplement: S2 File — (PDF) [file pone.0131468.s002.pdf]

심의면제 자가점검표

기 본 정 보						
연 구<br>과 제 명	반려견주와 반려견간 구강내 세균총의 비교	연 구<br>책 임 자	성 명	소 속	직 위	전공분야
			이중복	건국대학교	교수	수의학

다음은 심의면제가 가능한 경우를 점검합니다.(중복표시 가능)
1. 인간 또는 인체유래물을 대상으로 연구를 수행합니까?<br>☑ 예 (→ 2번 질문으로)<br>☐ 아니오 (→ 인간대상연구 또는 인체유래물연구의 심의 대상이 아닙니다.)
2. 다음에 해당한다면, 이 법의 적용 대상이 아닙니다. 해당하지 않는다면, 3번 질문으로 가세요.<br>① 국가나 지방자치단체가 공공복리나 서비스 프로그램을 검토·평가하기 위해 직접 또는 위탁하여 수행하는 연구<br>② 「초·중등교육법」 제2조 및 「고등교육법」 제2조에 따른 학교와 보건복지부장관이 정하여 고시하는 교육기관에서 통상적인 교육실무와 관련하여 하는 연구
3. 연구대상자 또는 인체유래물 기증자 및 공공에 미치는 위험이 미미합니까?<br>☑ 예 (→ 4번 질문으로) ※ 이 경우, 미미한 위험에 대한 판단은 연구자와 기관위원회가 일치해야만 하며, 불일치한다면 심의가 면제될 수 없습니다.<br>☐ 아니오 (→ 심의를 면제할 수 없습니다.)
4. 연구대상자 또는 인체유래물 기증자의 개인식별정보를 수집하거나 기록합니까?<br>☐ 예 (→ 심의를 면제할 수 없습니다.)<br>☑ 아니오 (→ 5번 질문으로)
5. 연구를 위해 연구대상자등에게 새로운 정보를 수집하지 않고 기존에 생성된 자료나 문서만을 이용하는 연구입니까?<br>☐ 예 (→ 심의를 면제할 수 있습니다.)<br>☑ 아니오 (→ 6번 질문으로)
6. 연구대상자 또는 인체유래물 기증자에 취약한 환경의 시험대상자가 포함되어 있습니까?<br>※ 취약한 환경의 시험대상자란 연구 참여와 관련하여 이익에 대한 기대 또는 참여를 거부하는 경우 조직 위계상 상급자로부터 받게 될 불이익에 대한 우려가 자발적인 참여 결정에 영향을 줄 가능성이 있는 연구대상자(학생, 의료기관·연구소의 근무자, 회사의 직원, 군인 등), 불치병에 걸린 사람, 집단 시설에 수용되어 있는 사람,

<table>
<tr><td colspan="4">실업자, 빈곤자, 응급상황에 처한 환자, 소수 인종, 부랑인, 노숙자, 난민, 미성년자 및 자유의지에 따른 동의를 할 수 없는 자를 말합니다.
☐ 예 (→ 심의를 면제할 수 없습니다.)
☑ 아니오 (→ 7-1번 질문으로)</td></tr>
<tr>
<td>7-1. 인간을 대상으로 연구를 위해 직접 어떤 조작이나 그의 환경을 조장하는 연구를 수행합니까?
☑ 예 (→ 8-1번 질문으로)
☑ 아니오 (→ 7-2번 질문으로)</td>
<td>7-2. 인간을 대상으로 면담, 설문조사 또는 행동관찰 등을 수행하여 얻은 자료를 이용하여 연구를 수행합니까?
☐ 예 (→ 8-2번 질문으로)
☑ 아니오 (→ 7-3번 질문으로)</td>
<td>7-3. 연구대상자(인간)를 식별할 수 있는 자료를 이용하여 연구를 수행합니까?
☐ 예 (→ 8-3번 질문으로)
☑ 아니오 (→ 7-4번 질문으로)</td>
<td>7-4. 인간을 직접 대상으로 하지 않지만, 인체로부터 얻어진 인체유래물을 직접 조사·분석하는 연구를 수행합니까?
☑ 예 (→ 8-4번 질문으로)
☐ 아니오 (→ 기관위원회로 문의하세요)</td>
</tr>
<tr>
<td>8-1. 다음의 어느 하나에 해당하면 심의를 면제할 수 있습니다.
☑ 약물투여, 혈액 채취 등 침습적 행위가 개입되지 않은 연구
☐ 신체적 변화가 초래되지 않는 단순접촉 측정장비 또는 관찰장비만을 사용하는 연구
☐ 식품위생법 시행규칙 제3조에 따라 판매 등이 허용된 식품의 맛 또는 질을 평가하는 연구
☐ 화장품법 제8조제1항 및 제2항에 따른 안전기준에 적합한 화장품을 이용하여 사용감 또는 만족도 등을 조사하는 연구</td>
<td>8-2. 다음에 해당하면 심의를 면제할 수 있습니다.
☐ 연구대상자가 불특정하며, 연구로 인해 수집된 정보에 개인정보보호법 제23조에 따른 민감정보가 포함되어 있지 않은 연구</td>
<td>8-3. 다음에 해당하면 심의를 면제할 수 있습니다.
☐ 일반 대중에게 공개된 정보를 이용하는 연구</td>
<td>8-4. 다음의 어느 하나에 해당하면 심의를 면제할 수 있습니다. 다만, 공중보건상 긴급한 조치가 필요한 상황에서 국가 또는 지방자치단체가 직접 수행하거나 위탁한 연구는 공용위원회에 연구 종료 전에 진행상황을 통보하여야 합니다.
☐ 인체유래물은행이 수집·보관하고 있는 인체유래물과 그로부터 얻은 유전정보(이하 “인체유래물등”)를 제공받아 사용하는 연구로서 인체유래물등을 제공한 인체유래물은행을 통하지 않으면 개인정보를 확인할 수 없는 연구
☐ 의료기관에서 치료 및 진단을 목적으로 사용하고 남은 인체유래물등을 이용하여 정확도 검사 등 검사실 정도관</td>
</tr>
</table>

			리 및 검사법평가 등을 수행하는 연구<br>☐ 인체유래물을 직접 채취하지 않는 경우로서 일반 대중이 이용할 수 있도록 인체유래물로부터 분리ㆍ가공된 연구재료(병원체, 세포주 등 포함)를 사용하는 연구<br>☑ 연구자가 인체유래물 기증자의 개인식별정보를 알 수 없으며, 연구를 통해 얻어진 결과가 기증자 개인의 유전적 특징과 관계가 없는 연구(다만, 배아줄기세포주를 이용한 연구는 제외한다)
